# Effectiveness of current protein recommendations in adolescent athletes on a low-carbon diet

**DOI:** 10.3389/fnut.2022.1016409

**Published:** 2022-09-15

**Authors:** Paula Albuquerque Penna Franca, Christine Katharine Alves Zago Gonçalves Lima, Taillan Martins de Oliveira, Tathiany Jéssica Ferreira, Renata Romanelli Mollini da Silva, Luiz Lannes Loureiro, Anna Paola Trindade Rocha Pierucci

**Affiliations:** Department of Basic and Experimental Nutrition (DNBE), Instituto de Nutrição Josué de Castro, Universidade Federal do Rio de Janeiro, Rio de Janeiro, Brazil

**Keywords:** adolescent athletes, environmental impact, food intake, protein recommended intake, water footprint, carbon footprint, planetary diet

## Abstract

Adolescent athletes require adequate energy and nutrient supply to support growth, development, and the demands associated with exercise and training. However, they are susceptible to nutritional inadequacies affecting their health and physical performance. Food choices with nutrient adequacy and environmental protection is crucial for a sustainable diet. Therefore, we aimed to assess the adequacy of low-carbon diets to meet the protein requirements of adolescent athletes. Therefore, a cross-sectional observational study was conducted with 91 adolescent athletes from sports clubs in Rio de Janeiro who underwent anthropometric and food consumption assessments. To estimate the environmental impact of anthropogenic activities, the sustainability indicators carbon footprint (CF) and water footprint (WF) were used. The CF of the athlete's diet was compared with the benchmark of 1,571 g CO_2_eq/cap/d estimated by the World Wildlife Fund (WWF). Protein recommendations according to the American Dietetic Association (ADA) for athletes and protein food groups according to the low-carbon EAT-Lancet reference diet were used as references. The results were stratified by sport modality, age, sex, and income range. The Mann-Whitney test was performed, followed by the Kruskal-Wallis test with Dunn's *post-hoc* test to assess the differences between groups using the statistical program GraphPad PRISM® version 8.0. CF and WF were directly associated with total energy intake, total protein intake, animal-origin protein intake, and the food groups of meat and eggs. Significant differences were observed in the environmental impact of diet based on sports groups and gender. The athletes' profile with higher environmental impact was male, middle-income class, and of any age group. The quartiles of CF of the overall diets were above the 1,571 g CO_2_eq/cap/d benchmark. Additionally, ADA's recommended range of daily protein consumption was met by most athletes, even in the lowest quartile of CF. Thus, a diet with a lower environmental impact can meet protein recommendations in adolescent athletes. The results found are of interest to the sports and food industries. It could help in designing a balanced diet for athletes as well as ensure less negative environmental impacts of food production and consumption.

## Introduction

Athletes' diets are generally high enough in protein, if not excessive (1). The American dietetic association (ADA) (2) recommends a protein intake of 1.2–2.0 g per kilogram (kg) of body mass for adult athletes, from good quality protein sources divided into moderate portions throughout the day. However, it is a common belief among athletes that sources of animal protein are needed for muscle recovery (3) and that meat bioactive molecules have functional properties that exert positive effects on athletic performance and overall health status of athletes (4). However, ruminant products, including meat and dairy, have the highest greenhouse (GHG) gases emissions per kilogram, per kilocalorie, per serving, due to methane from the enteric fermentation and nitrous oxide from manure (5).

According to Jeukendrup and Cronin (6) young athletes have a high intake of protein above the recommended daily intake (RDA). Additionally, (7) more than 55% of adolescent athletes of both sexes (*n* = 129) practicing tennis, swimming, gymnastics (artistic and rhythmic), and judo have been reported to have excessive intake of carbohydrates and proteins. Higher protein consumption is likely to increase the demand for animal protein production, including meat, dairy, and eggs. As meat production increases, greater areas of native biomes are devastated due to the increased need of pastures or the production of cattle feed (8).

In Brazil, ~80% of the deforestation in the Amazon Forest is due to livestock activities (9). Moreover, deforested land is often cleared using fires emitting GHGs. In addition to contributing to GHG emissions, deforestation causes environmental degradation and natural habitat destruction, causing the extinction of native species. Furthermore, it causes soil degradation and erosion triggering multitudes of issues including floods, compromised water resources, and changes in rainfall regimes (8).

Different environmental indicators can be used to assess the environmental impacts of food production and consumption (10). GHG emission, also called carbon footprint (CF), is associated with global warming potential and is widely discussed while addressing responsibility and mitigation of global warming (11). CF measures the total amount of GHG gas emissions (CO_2_, CH_4_, N_2_O, HFC, PFC, and SF_6_) over the entire life cycle of a product, food, event, individual, and so on. Another widely used indicator is the water footprint (WF). Based on the latest IPCC report (12), the World Wildlife Fund (WWF) (13) has set a per capita dietary food footprint of a maximum of 11 kg CO_2_ eq/week to keep global warming below 1.5°C. The WF has been used as an indicator of the water consumption of people and products in different parts of the world (14–16).

In 2019, the EAT Lancet Commission on Healthy Diets from Sustainable Food Systems published a new global sustainable diet benchmark to promote human health, while remaining within the planet's ecological boundaries. The commission concluded that a change to plant-based diets has a high potential for mitigating global warming (17). The report also highlights the need for a major shift in our eating habits to keep the food system within sustainable boundaries (17). However, the potential of the EAT-Lancet reference diet for application in the diets followed by the general population is unclear, as it was conceived as a general reference for a sustainable planetary diet (18).

Recently, studies focusing on integration of athletes' dietary choices with sustainable food systems have been published (1, 19–22). However, to date, no studies have estimated the environmental impact of diet of adolescent athletes. Therefore, the work aimed to analyze food, energy, and protein intake, concerning CF, WF and EAT-Lancet reference diet (17) among adolescent athletes from different Olympic modalities. This study intends to provide better nutritional guidance compatible with nutritional demands and lower environmental impacts. This is especially relevant in the context of conservation of forest and biodiversity being exploited to maintain the population's high consumption of all types of meat and dairy products (23).

## Materials and methods

### Study design and participants

This cross-sectional study included 91 adolescent athletes aged 11–19 years, who were practitioners of the Olympic modalities: judo, swimming, water polo, and artistic swimming. The study was approved by the Research Ethics Committee of Clementino Fraga Filho University Hospital/UFRJ CAAE 58179716.3.0000.5257. The participants and their guardians were informed about the procedures and risks involved and gave their written consent.

### Socioeconomic data

All participants completed a questionnaire on demographic and socioeconomic data (age, sex, and monthly family income). For the classification of monthly family income, the national criteria adopted by population surveys stratified by minimum wages were used (24): Level 1, from 1 to 2 minimum wages; Level 2, from 2 to 4 minimum wages; Level 3, from 4 to 10 minimum wages; Level 4, from 10 to 15 minimum wages; and Level 5, above 15 minimum wages.

### Anthropometry

Weight was measured using a Filizola® 150 kg digital electronic scale balance with a maximum load of 150 kg, minimum load of 2.5 kg and sensitivity of 0.1 kg (Campo Grande, Brazil). Height was measured using an Alturexata Stadiometer® with bilateral wooden ruler scaled in millimeters (1 mm resolution) and field of use from 0.35 to 2.15 mm (Belo Horizonte, Brazil). Both measurements were performed in duplicate. From these data, body mass index (BMI = kg/m^2^) was calculated. The participants' weight was evaluated according to BMI, sex, and age (25).

### Food intake

Food consumption was evaluated using a 24-h food record. Participants were asked to detail the food consumed, specifying quantities, time of ingestion, portion sizes, and brands of products consumed the day before the interview (26). Nutritional supplements were also analyzed as they contribute to daily nutrient and energy intake (2). For the quantitative analysis of nutrients and energy, data were converted to International System units using a table for assessment of food consumption in household measures (27). Protein consumption was evaluated based on the recommendations of the American Dietetic Association (ADA) (2), which recommends 1.2–2.0 g/kg of body weight/day protein consumption based on the level of physical activity (moderate to intense). We also stratified protein intake by animal protein and plant-based protein to evaluate the effect of origin of the protein on environmental indicators.

### Comparison of food consumption with EAT-Lancet reference diet

The Eat-Lancet reference diet is based on a 2,500 kcal diet; foods are divided into eight food groups, and protein-rich foods are divided by source into six subgroups. This study analyzed food groups that are protein sources and dairy products. For analysis, the percentage of the energy contribution of each food group was calculated (Table 1). Subsequently, the percentage (%) of the energy contribution of each protein food group reported by the study participants was compared to the % of energy contribution recommended by the EAT-Lancet Commission (17).

**Table 1:** EAT-Lancet reference diet in energy and percentage contribution to total energy intake for protein source food groups.

	**Energy intake/kcal per day**	**Percent contribution to total energy intake**
Dairy foods	153	6.11
Animal protein		6.03
Red meat	30	
Poultry	62	
Eggs	19	
Fish	40	
Plant-based protein		22.97
Nuts	284	
Legumes	291	

### Estimation of the environmental impact of the diet

The environmental footprints of the foods consumed were calculated based on the database developed by Garzillo et al. (28), which provides the CF and WF coefficients. Furthermore, the authors reviewed primary data on footprints, including scientific publications and industrial reports, prioritizing primary data from Brazilian food production. The different cooking methods were also included in the analysis to consider CF and WF “from farm to fork.” CF represents the amount of GHGs emitted by food intake (g CO_2_eq/cap/d), and WF is the sum of three specific components: green, blue, and gray water (liters/cap/d). Additionally, the upper limit of 1,571 g CO_2_eq/cap/d of CF proposed by the WWF (2021) (13) was compared with the CF of the daily diet set to keep global warming below 1.5°C (12).

### Statistical analysis

The Kolmogorov-Smirnov test was used to assess data normality. The data showed a non-parametric distribution; therefore, they were expressed as median, minimum, and maximum. The study variables were compared by sex, age group, income level, and type of sport using the Mann-Whitney test, followed by the Kruskal-Wallis test with Dunn's *post-hoc* test for comparisons between the groups. The correlation between the variables and environmental impact of the diet was evaluated using Spearman's correlation. The association between footprints and the variables were evaluated using an n-path analysis. All analyses were performed using GraphPad PRISM® version 8.0. The power of the test was calculated a posteriori based on a mean effect size of 0.15, for a sample of 91 participants. The calculations showed that at a significance level of 5%, the statistical power was equivalent to 87%. G-Power® software (version 3.1.9.2) was used.

## Results

The final sample consisted of 91 confederate athletes categorized according to age group, sex, sports modality, and family monthly income level. The median total mass, height, and body mass index found were 56.8 kg (34.4–97.7), 1.62 m (1.40–1.81), and 21.3 kg/m^2^ (16.1–33.5), respectively. As for body composition, we observed that most of the participants (67.0%, *n* = 61) were classified as eutrophic, with an average BMI of 21.6 kg/m^2^, 28.6% (*n* = 26) were overweight and 4.4% (*n* = 4) were obese. Statistical differences were observed between age groups for height (*p* < 0.0001), body mass (*p* < 0.0001), and BMI (*p* < 0.0001) and between sex, for height (*p* = 0.0012), and body mass (*p* = 0.0223). Anthropometric and socioeconomic results are shown in Table 2.

**Table 2:** Anthropometric data, energy intake and protein intake of the adolescent athletes participating in the research, categorized by age group, gender, sport modality and monthly family income levels.

	**Total**	**Age group**		**Gender**		**Sport modality**				**Family income levels**				
	**(*n* = 91)**	**11–14 years** **(*n* = 52)**	**15–19 years** **(*n* = 39)**	**Female** **(*n* = 45)**	**Male** **(*n* = 46)**	**Judo** **(*n* = 36)**	**Artistic swimming** **(*n* = 21)**	**Swimming** **(*n* = 25)**	**Water Polo** **(*n* = 9)**	**1** **(*n* = 14)**	**2** **(*n* = 16)**	**3** **(*n* = 31)**	**4** **(*n* = 20)**	**5** **(*n* = 10)**
Heigh (cm)	162.0 (140.0–181.0)	158.0 (140–174)^*^	166.7 (149–181)^*^	159.8 (144–171)^*^	166.3 (140–181)^*^	162 (140–176.5)	160.6 (145–170.2)	165 (144–181)	167 (150–181)	159.5 (140–175)	163 (145–179)	165 (146–176)	158.2 (140–181)	159.9 (144–181)
Body weight (Kg)	56.8 (34.4–97.7)	51.8 (34.4–97.7)^*^	62.7 (45.2–93)^*^	53.8 (34.4–84.3)^*^	59.9 (34.6–97.7)^*^	56.0 (34.0–93.0)	54.6 (39.1–65.3)	57.5 (38.3–84.4)	63.5 (42.6–97.7)	57 (36.6–67.2)	60.5 (39.1–72.9)	57 (34.4–97.7)	53.8 (34.6–84.3)	52.3 (39.6–84.4)
BMI (kg/m^2^)	21.3 (16.1–33.5)	20.3 (16.1–32.3)^*^	22.7 (19.3–33.5)^*^	20.9 (16.1–32.1)	21.5 (16.1–33.5)	21.6 (16.1–33.5)	20.7 (16.3–25.2)	21.1 (17.2–25.7)	22.6 (18.9–32.3)	22.1 (16.1–24.8)	22.8 (18.4–25.5)	21.4 (16.1–33.5)	20.3 (17.2–25.7)	20.8 (18.8–25.7)
Protein intake (g/kg)	1.8 (0.4–3.8)	1.8 (0.5–3.8)^*^	1.6 (0.4–3.3)^*^	1.5 (0.6–3.1)^*^	1.8 (0.4–3.8)^*^	1.5 (0.4–3.8) ^a^	1.3 (0.7–2.7) ^b^	2.0 (1.3–3.1) ^a^	1.5 (0.6–3.3) ^a^	1.5 (0.8–2.3)	4.3 (2.4–8.2)	1.9 (0.5–3.8)	1.6 (0.6–3.1)	1.9 (1.1–2.8)
Calorie intake (kcal/day)	1,953.4 (1,030.2–3,389.1)	1,938.4 (1,030.2–3,304.7)	1,967.4 (1,125.5–3,389.1)	1,769.6 (1,030.2–3,304.7)	2,160.1 (1,280.3–3,389.1)	1,960.4 (1,265.7–3,349.9)^a^	1,571.1 (1,030.2–2,067.8)^b^	2,215.8 (1,398.3–3,389.1)^a^	2,100.1 (1,867.9–2,761.3)^a^	1,769.8 (1,280.3–2,205.9)	1,882.4 (1,215.0–3,349.9)	1,902.5 (1,125.5–3,159.3)	2,063.3 (1,101.4–3,304.7)	2,131.1 (1,030.2–3,389.1)

Data are expressed as median, minimum, and maximum; centimeters; kg- kilograms; BMI- body mass index; kg/m^2^- kilograms per square meter; family income levels: 1- up to 2 minimum wages or up to R$ 1,449.99; 2- from 2 to 4 minimum wages or between R$ 1,500.00 and R$ 2,899.99; 3- from 4 to 10 minimum wages or between R$ 2,900.00 and R$ 7,249.99; 5: from 10 to 15 minimum wages or between R$ 7,250.00 and R$ 14,499.99; 6: above 15 minimum wages or above R$ 14,500.00. Values not sharing superscript letters are different at the univariate level, *p* < 0.05.

* Significant difference between categorized groups (*p* < 0.05).

**Figure 1:**
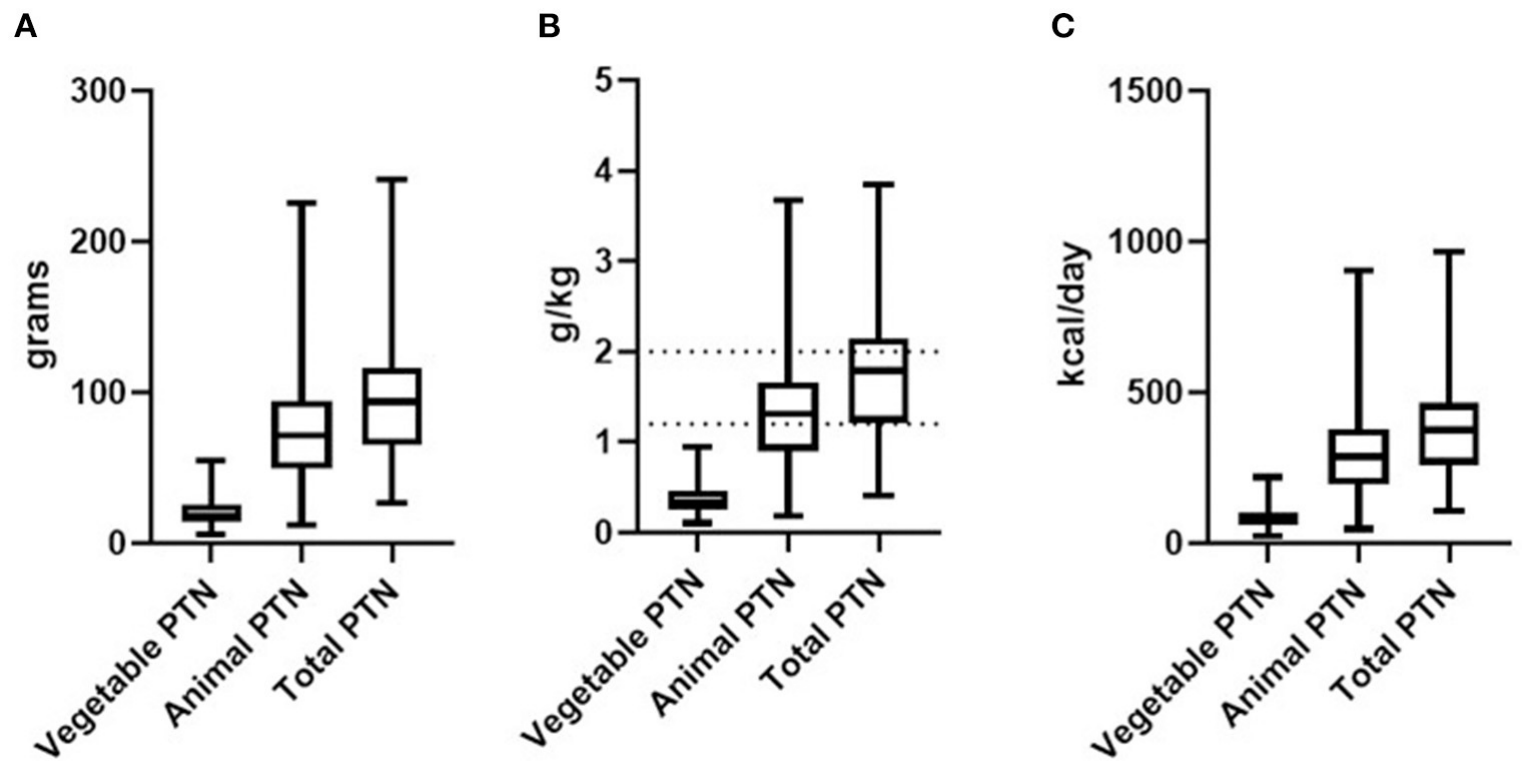
Daily protein intake (g) **(A)**, daily protein intake per kilogram of body weight (g/kg) and the ADA (2) recommendation range for protein (1.2–2.0 g/kg/day) **(B)**, and daily energy intake from protein **(C)** of adolescent athletes according to the food source of protein. Data expressed as median, minimum, and maximum. Dotted lines: Recommendation range (1.2–2.0 g/kg).

The protein intake of the participants was 1.78 (0.4–3.8) g of protein per kilogram of body weight (g/kg). The median amount of vegetable protein was 0.34 g/kg of body weight, while animal protein was 1.31 g/kg. Artistic swimming participants' protein intake [1.3 (0.6–2.7) g/kg of body weight] was lower (*p* = 0.0061) than that of swimming participant's [2.0 (1.3–3.1) g/kg of body weight]. While protein intake for judo participants was 1.5 (0.4–3.8) g/kg of body weight, and water polo participants was 1.5 (0.6–3.3). The protein intake was within the ADA's (2) recommended range for 49.4% (*n* = 45) of the participants, while 27.4% (*n* = 25) had higher protein intake, and 23% (*n* = 21) had lower intake than the reference values. Additionally, the participants did not report using nutrition and protein supplements. The protein intake according to food source, total energy from protein intake/day and ADA's (2) recommendation range is represented in Figure 1.

Table 3 shows the CF and WF values of the reported diet of the participants according to age, sex, and sociodemographic variables. The median of the entire group for CF and WF was 4,999.5 g CO_2_eq/cap/d (1,379.3–18,668.1), and 4,943.6 liters/cap/d (1,222.9–12,829.3), respectively. The athlete group with the greatest environmental impact was male. The only sports modality that presented differences in CF and WF from the other modalities was artistic swimming (*p* < 0.0001).

**Table 3:** Carbon and water footprints of adolescent athletes' diet, according to age groups, gender, sport modality, and monthly family income levels, expressed as median, minimum and maximum.

		**Age group**		**Gender**		**Sport modality**				**Family income level**				
	**Total** **(*n* = 91)**	**11–14** **years** **(*n* = 52)**	**15 −19 years** **(*n* = 39)**	**Female** **(*n* = 45)**	**Male** **(*n* = 46)**	**Artistic swimming** **(*n* = 21)**	**Water polo** **(*n* = 9)**	**Swimming** **(*n* = 25)**	**Judo** **(*n* = 36)**	**1** **(*n* = 14)**	**2** **(*n* = 16)**	**3** **(*n* = 31)**	**4** **(*n* = 20)**	**5** **(*n* = 10)**
Carbon footprint (g CO_2_ eq)	4,999.5 (1,379.3–18,668.1)	4,131.4 (1,379.3–12,471.0)	5,722.2 (1,399.3–18,668.1)	3,804.5 (1,399.3–18,668.1)^*^	5,291.2 (1,379.3–17,280.8)	2,656.4 (1,399.3–7,635.4)^**^	5,298.1 (2,378.1–14,630.5)	6,175.6 (2,527.4–17,280.8)	4,788.6 (1,379.3–18,668.1)	4,041.9 (1,691.9–18,668.1)	4,763.4 (1,399.3–14,508.8)	5,160,0 (1,444.3–14,630.5)	4,442.1 (1,379.3–12,159.7	4,606.7 (1,991.4–17,280.8)
Water footprint (liters)	4,943.6 (1,222.9–12,829.3)	4,835.9 (1,626.0–11,562.6)	5,887.2 (1,222.9–12,829.3)	4,394.0 (1,518.1–12,829.2)^*^	6,562.4 (1,222.9–12,672.6)	3,350.9 (1,530.1–11,255.8)^**^	8,812.9 (4,413.5–11,608.6)	5,962.8 (2,733.1–12,672.6)	6,208.6 (1,222.9–12,829.2)	3,849.0 (1,632.8–12,829.2)	4,449.8 (1,222.9–12,163.6)	5,613.5 (1,530.1–11,255.8)	4,803.2 (1,626.0–11,562.6	5,287.4 (2,733.1–12,672.6)

Data are expressed as median, minimum, and maximum; gCO2 eq: carbon dioxide equivalent; m2: meter squared; family income: 1–2 minimum wages or up to R$ 1,449.99; 2- from 2 to 4 minimum wages or between R$ 1,500.00 and R$ 2. 899.99; 3- from 4 to 10 minimum wages or between R$ 2.900,00 and R$ 7.249,99; 4- from 10 to 15 minimum wages or between R$ 7.250,00 and 14.499,99; 5–above 15 minimum wages or above R$ 14.500,00.

* Significant difference between gender;

** Significant difference in artistic swimming in relation to the other sports modality.

The energy and protein intake and environmental footprints across quartiles of CF are represented in Table 4. The CF values were above the benchmark of 1,571 g CO_2_eq/cap/d for a diet compatible with global warming below 1.5°C. The protein median was within the range recommended by the ADA (2) for the four quartiles. No statistically significant difference was observed in vegetable protein intake among quartiles. Animal protein levels differed from the 1st to 4th quartile.

**Table 4:** Energy and protein intake and environmental footprints across quartile of carbon footprint.

	**Q1**	**Q2**	**Q3**	**Q4**
Carbon footprint	2,009 (1,379–2,704)^a^	3,710 (2,751–5,000)^b^	6,206 (5,002–7,762)^c^	10,643 (7,890–18,668)^d^
Water footprint	2,627 (1,223–6,697)^a^	3,840 (1,633–8,497)^a, b^	5,306 (4,077–1,1256)^b, c^	9,106 (5,614–12,829 ^d^
Energy (day)	1,571 (1,030–2,913)^a^	1,934 (1,259–3,159)^a, b^	2,011 (1,354–2,765)^b, c, d^	2,440 (1,398–3,389)^c, d^
Protein (g/kg/day)	1.2 (0.4–2.6)^a^	1.5 (0.6–3)^a, b^	1.7 (0.7–2.8)^a, b^	2.0 (0.9–3.8)^b^
Vegetable protein (g/kg/day)	0.31 (0.1–0.6)	0.37 (0.14–0.91)	0.43(0.17–0.84)	0.31(0.17–0.94)
Animal protein (g/kg/day)	0.9 (0.18–2)^a^	1.16(0.4–2.6)^a, b^	1.28(0.51–2.1)^a, b^	1.59 (0.75–3.67)^b^

Values are expressed as median, minimum, and maximum. Data were analyzed using the Kruskal Wallis test with Dunn's correction; values not sharing superscript letters are different at the univariate level, *p* < 0.05.

**Figure 2:**
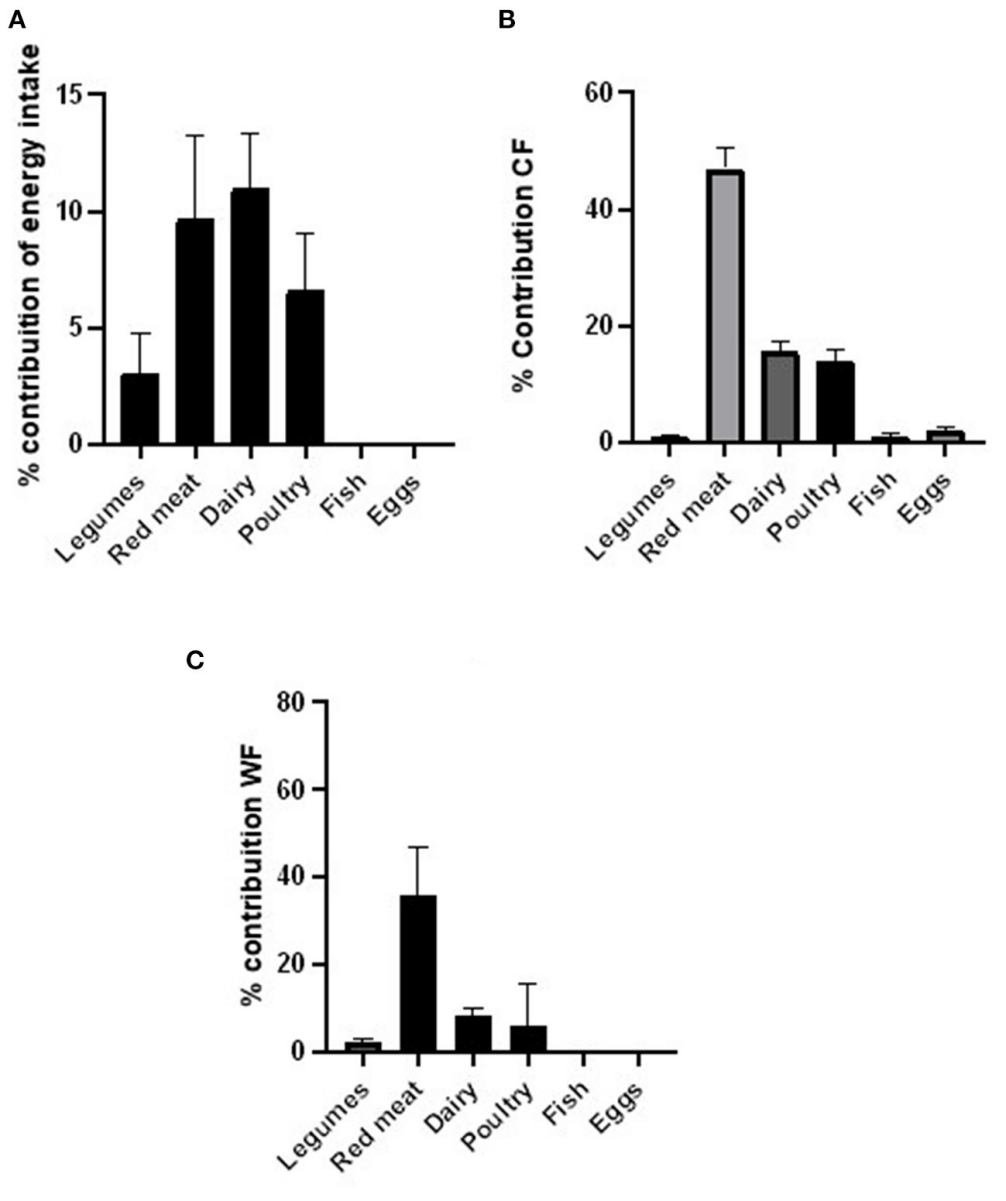
Percentage contribution from Eat-Lancet reference diet food groups to energy intake **(A)**, carbon footprint **(B)**, and water footprint **(C)**.

**Figure 3:**
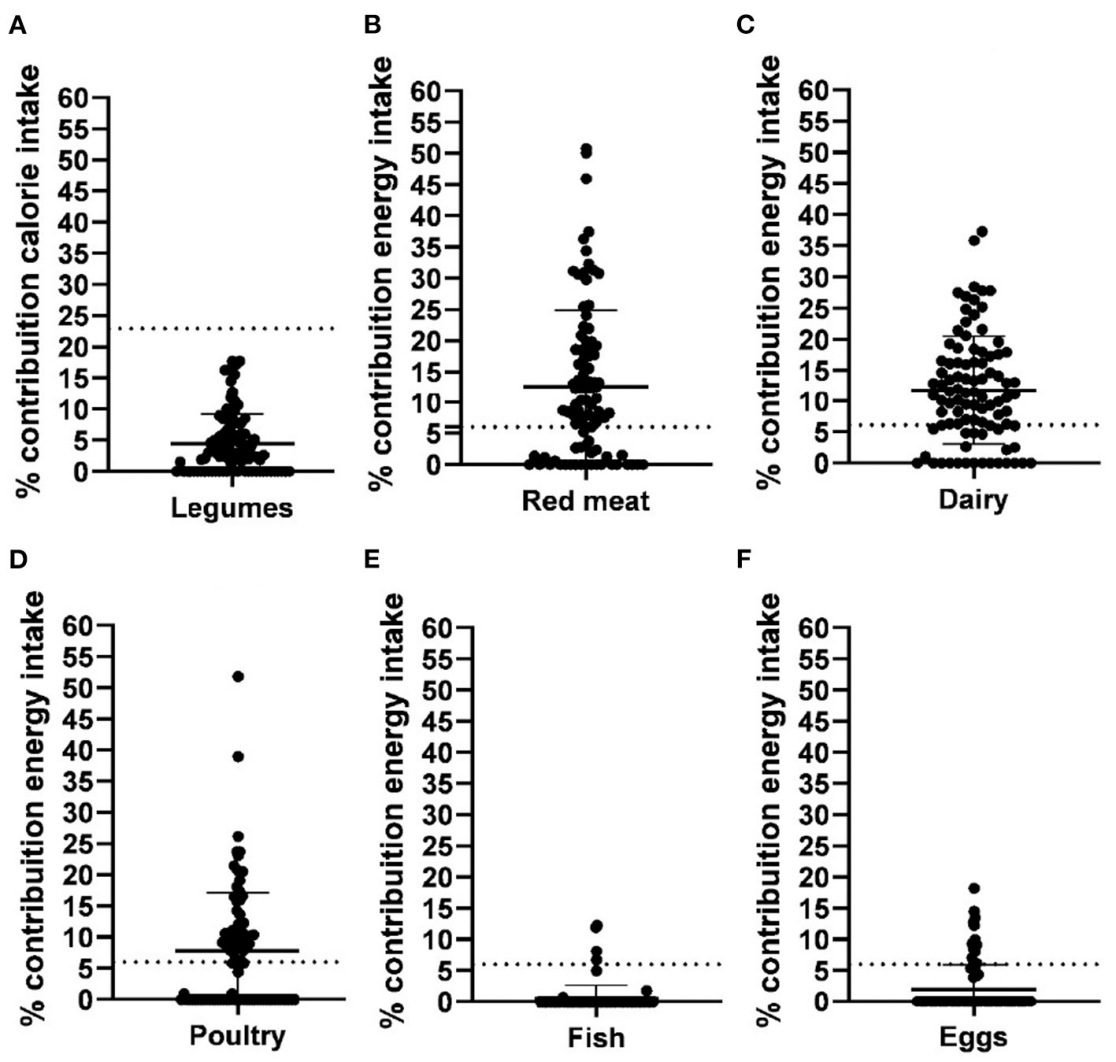
Comparison of the percentage contribution of energy intake by protein food groups of adolescent athletes compared to percentage % contribution of energy intake by protein food groups of the EAT- Lancet reference diet. Y axis: percentage contribution of energy intake from the food group; Dotted lines: EAT- Lancet reference diet for food group. Legumes and nuts **(A)**; Red meat **(B)**; Dairy **(C)**; Poultry **(D)**; Fish **(E)**; Eggs **(F)**.

The percentage contributions of each of the six protein groups in the EAT-Lancet reference diet to energy intake, CF, and WF are shown in Figure 2. The percentage contribution of animal protein food groups to energy intake was 33.6%. Animal protein food groups contributed to 85.9% of the CF and 78.4% of WF in the reported diets. A comparison between the EAT-Lancet reference diet and participants' intake is shown in Figure 3. Values are expressed as the percentage of energy contribution for each food group. The ingestion of legumes and seeds was below the recommended levels for all participants, and the fish intake was below the recommended levels as per the Eat-Lancet reference diet for most participants. Red meat, dairy, and poultry intake was above the recommended levels as per the EAT-Lancet reference diet.

## Discussion

The present study estimated the environmental impact of food intake of adolescent athletes from different Olympic modalities, using CF, WF, and Eat-Lancet as a reference to evaluate the association of recommended protein intake and sustainable food consumption (17). Additionally, the participants underwent anthropometric assessment and were classified according to their socioeconomic conditions. The results showed that food intake and the environmental impact of diet varied according to the practice modality. CF and WF were moderately correlated with intake of animal protein, total protein, red meat, and eggs.

BMI is not the best indicator for evaluation athletes' health, as it does not consider the distribution of body fat (29). However, in the case of adolescent participants, this indicator becomes relevant for assessing their growth and development (29, 30). In the present study, 26 participants were overweight. Additionally, three of the ten judo practitioners were obese. These results are supported by existing literature, including the study by Sotoriva and Miraglia (31), which evaluated Brazilian Judo athletes, with a mean age of 15.6 ± 1.6, BMI of 23.3 ± 2.5 kg/m^2^ and reported that 69.2% of the participants were eutrophic and 30.8% of them were overweight based on BMI.

Of the participants, 41.8% (*n* = 45) could meet the protein recommendations (2). However, 35.0% (*n* = 32) of the participants consumed protein above the recommendation level. This high consumption of protein may be due to the desire to increase muscle mass; however, consumption above the recommended level of protein does not increase protein synthesis; therefore, it is not useful (32). For the ingested protein to be used for protein synthesis instead of oxidation for energy supply, adequate energy intake is important, especially from carbohydrates (2).

Current data suggest that the dietary protein intake required to support metabolic adaptation, repair, remodeling, and protein turnover generally range from 1.2 to 2.0 g/kg/day (2). The daily protein intake goals should be met with a meal plan that provides a regular distribution of moderate amounts of high-quality protein throughout the day and after strenuous training sessions. These recommendations cover most training regimens and allow for flexible adjustments with periodic training and experience (33). Regarding the total protein intake of the participants, consumption of animal protein (78%) was almost four times higher than that of plant-based proteins such as legumes.

Regarding the Eat-lancet reference diet, all animal protein food groups, except for fish, consumed by the participants were higher than the recommended level. However, as the Eat-Lancet is a universal recommendation, it is necessary to adapt the EAT-Lancet reference diet to national preferences and contexts, as well as local food availability, nutritional content of foods, and national dietary recommendations (34). The CF and WF relative to Eat-Lancet reference food groups were not surprising, as animal protein is known to have a high environmental impact, high CF and WF as compared to that of plant protein (35). This is especially important because, in Brazil, there is a tradition of appreciating meat, and people consider animal protein essential for health and nutrition (36). However, such a high intake of dairy was not expected, which might be associated with adolescent consumption of yogurts, sandwiches, and pizza, which are very common in this age group (24). We expected to observe a higher intake of legumes, as beans are a staple food in Brazil and are traditionally consumed twice a day along with rice, vegetables, and a source of meat (37, 38). Beans were the second-most consumed food (142.2 g/person/day) reported at *Pesquisa de Orçamentos Familiares* 2018–2019 (POF—Household Budget Survey 2018–2019), while the participants in our study reported a median intake of 70 g (24).

No association was found between income levels and food groups or between income levels and CF and WF. Meat consumption may vary according to income, increasing proportionally to income until it reaches a plateau and then drops slightly (39). On the other hand, it is expected that people with greater purchasing power will be more aware about sustainability. Therefore, this group would be more susceptible to adopting a diet with low environmental impact (40).

In our study, male participants had higher CF, WF, and energy intakes than female participants. It is well-documented that footprints are associated with energy intake (35). The older age groups also had higher footprints than the younger group, which is probably due to the higher energy intake among older athletes. Maintaining a diet within the energy recommendations is a possible way to stimulate healthy and sustainable diets (35). However, athletes have a high total energy expenditure, and this procedure is not suitable for athletes. Among the different sports modalities, artistic swimming participants had lower values of CF and WF; these athletes also had lower energy intake, and in the group studied, all were female. Therefore, the lower environmental impact of this modality is likely associated with sex and lower energy intake.

Garzillo (10) evaluated the environmental impact (CF and WF) of average Brazilian food consumption based on data from the *Pesquisa de Orçamentos Familiares* 2008–2009 (POF—Household Budget Survey 2008–2009). An estimated value of 4,130 g CO_2_eq/cap/d was obtained for CF, where red meat represented 56.0% of the average dietary carbon emissions. The estimated WF value is 4,124 liters of water per day. The average energy intake was 1,977 kcal and the median protein intake was 76 g. Red meat represented 56.6% of the total value of CF. Regarding WF, red meat's contribution was 49.2%, followed by legumes (7.5%), fruits (3.3%), and vegetables (3.1%). In comparison with the study carried out by Garzillo (10), the adolescent athletes in our study had a higher WF (4,943.6 liters) and CF (4,999.5 g CO_2_eq/cap/d). Meat's contribution to WF and CF, 78.4 and 85.9%, respectively, can also be considered high.

Other studies have used higher estimations of CF for a sustainable diet: 1,337 kg CO_2_eq/cap/yr, equivalent to 3,713 g CO_2_eq/cap/d (41), and 3,288 g CO_2_eq/cap/d for a healthy sustainable diet (42). If these values were used, the diet of participants in the first quartile of CF could be considered sustainable. However, the other quartiles were high-intensity emissions. CF estimates for the fourth quartile were superior to the average dietary emissions from high-emission countries such as the USA (43).

The different methodologies used to estimate the environmental impact of food consumption make it difficult to compare results between studies. The food consumption of adolescent athletes presented in this study had higher values for CF (4,999.5 g CO_2_eq/cap/d) than in the studies carried out by Travassos et al. (44), Reguant-Closa et al. (21), and Arrieta and Gonzáles (45). The values found for WF (4,943.6 L) can be considered high compared to the results of Travassos et al. (44). However, the footprint database used by these authors has a different methodology than that used in the present study because they did not consider the cooking techniques. Travassos et al. (44) analyzed the contribution of the Brazilian diet to CF and WF based on food consumption data from the POF 2008–2009. The mean values found for Brazilian adults (*n* = 24,152) for energy intake were 1,578.2 ± 569.9 kcal/day; CF was 6,760.6 ± 4,920.7 g CO_2_eq/cap/d, and the WF was 3,476.4 ± 1,830.8 L/d. Arrieta and Gonzáles (45) estimated that GHG emissions related to the current diet in Argentina are 5.48 kg CO_2_eq/cap/d. The CF found in this study was considered high, with foods of animal origin contributing to the largest share of emissions (71%). Temme et al. (46) evaluated the CF of 1 day of food consumption in the Netherlands in boys and girls (7–18 years). Girls had an average of 3.2 kg CO_2_eq of CF, with an energy intake of 2,015 kcal/day, and for boys 3.7 kg CO_2_eq, with an energy intake of 2,3960 kcal/day. Approximately 40% of the GHGs in the diets came from meat and cheese, and the contribution of beverages was ~20%, represented by dairy drinks, soft drinks, coffee, and tea.

A study carried out in Sweden used linear programming to design nutritionally adequate and climate-friendly diets for omnivorous, pescatarian, vegetarian, and vegan adolescents (47). The results showed that an affordable and nutritionally adequate diet with considerably reduced GHG can be achieved for adolescents. For omnivorous adolescents, the diet was associated with a reduction in meat, dairy, and processed foods, and an increase in cereals and tubers, pulses, eggs, and seafood (47).

We observed a considerable disproportion between the percentage of energy contribution (33.6%) and the percentage contribution of CF (85.9%) and WF (78.4%) from animal protein. In the first quartile, the plant-to-animal protein was 1:3. In the quartile with the highest CF, the ratio of plant-to-animal protein was 1:5, reinforcing the need to reduce animal protein consumption to decrease the environmental impact of the diet. Reducing the intake of animal protein and reducing energy intake should be ranked as the first and second steps to steer current food consumption in a sustainable direction, and reducing household food waste should be ranked third (48).

Therefore, it is evident that vegetarian and vegan diets do not impair sports performance. Lynch (19) compared vegetarian and omnivorous endurance athletes and found that their ability to generate strength was equivalent, and vegetarians showed better cardiorespiratory fitness than omnivorous athletes. Nebl et al. (49) found similar results when comparing the exercise capacities of vegan, lacto-ovo-vegetarian, and omnivorous athletes; the three groups presented the same capacity. Thus, it is possible to note that adopting a vegetarian diet does not bring any harm to sports performance, and there are still benefits to the health of these individuals reducing the risk of developing chronic diseases (50). It is worth mentioning that for athletes to be able to reduce the environmental impact of their diet, they do not need to become vegetarian or vegan. Reguant-Closa et al. (20) highlighted in their study that replacing part of animal protein with plant-based protein already leads to significant mitigation of environmental impacts.

There are several ways to introduce sustainable strategies in sports nutrition, such as adopting a flexible diet with a greater intake of plant-based protein than animal protein, not exceeding the daily protein intake needs, reducing the consumption of animal protein, reducing food waste, prioritizing the production of local and seasonal foods, reducing the consumption of processed foods, and prioritizing sustainable packaging. These guidelines can aid in reconciling sports nutrition, performance, and food sustainability. However, nutritional recommendations that consider sustainability are still scarce (1, 22, 51). Therefore, it is important to reinforce the scientific evidence of the environmental impact of the diet of athletes, especially adolescents, which highlights the importance of the present study, since adolescent athletes have different consumption patterns and nutritional needs compared to other groups.

To the best of our knowledge, no previous study has calculated the environmental impact of the diets of adolescent athletes. This area of research needs further studies to provide insights into the environmental boundaries of diets, food and nutrient intakes and sports performance. We also emphasize the importance of providing nutritional guidance for sustainability. Discussions about sustainable diets are still very incipient in Brazil, and different initiatives and clarification campaigns are necessary to encourage the reduction of animal protein consumption.

Our results demonstrate that Brazilian adolescent athletes' food intake was above the benchmark of 1,571 g CO_2_eq/cap/day for a diet compatible with global warming below 1.5°C. The median of the protein was within the range recommended by ADA (2), even in the lowest quartile of CF, which demonstrates that it is viable to have a lower environmental impact while meeting the protein recommendations.

Furthermore, our findings show some discrepancies between the diet of athletes and the reference planetary diet of the EAT-Lancet. This may be explained by the fact that the EAT-Lancet reference diet was conceived as a recommendation for the general population and not for specific groups. Therefore, it could be used as a roadmap and is not strictly followed by athletes because it has many stress points. Athletes have higher protein and energy recommendations than the general population. Although athletes can follow a plant-based diet, nutritional guidance is not always available.

Our results demonstrated that it is possible to have athletes on a low-carbon diet and simultaneously meet protein recommendations, with a plant-to-animal protein ratio of 3 × 1. However, they also had a lower calorie intake, which can compromise sports performance; therefore, future research should evaluate athletes' diets in terms of sustainability and sports performance.

## Data availability statement

The original contributions presented in the study are included in the article/Supplementary material, further inquiries can be directed to the corresponding author/s.

## Ethics statement

The studies involving human participants were reviewed and approved by Research Ethics Committee of the Clementino Fraga Filho University Hospital/UFRJ CAAE 58179716.3.0000.5257. Written informed consent to participate in this study was provided by the participants' legal guardian/next of kin.

## Author contributions

PF and CG: conceptualization, methodology, investigation, writing—original draft, and visualization. TO: formal analysis, investigation, writing—original draft, and writing. TF: writing—review and editing. LL: formal analysis, investigation, writing—original draft, and editing. RS: writing—original draft. AP: conceptualization, methodology, resources, writing—review and editing, project administration. All authors contributed to the article and approved the submitted version.

## Funding

This study was funded by the Fundação Carlos Chagas Filho de Amparo a Pesquisa no Estado do Rio de Janeiro FAPERJ process E-26/201.042/2021.

## Conflict of interest

The authors declare that the research was conducted in the absence of any commercial or financial relationships that could be construed as a potential conflict of interest.

## Publisher's note

All claims expressed in this article are solely those of the authors and do not necessarily represent those of their affiliated organizations, or those of the publisher, the editors and the reviewers. Any product that may be evaluated in this article, or claim that may be made by its manufacturer, is not guaranteed or endorsed by the publisher.

## References

[1] Meyer N Reguant-Closa A. Eat as if you could save the planet and win! Sustainability integration into nutrition for exercise and sport. Nutrients. (2017) 9:412. 10.3390/NU9040412 28430140 PMC5409751

[2] Thomas D Erdman KA Burke LM. American college of sports medicine joint position statement. Nutr Athletic Perform Med Sci Sports Exerc. (2016) 48:543–68. 10.1249/MSS.0000000000000852 26891166

[3] Eck KM Byrd-Bredbenner C. Food choice decisions of collegiate division i athletes: a qualitative exploratory study. Nutrients. (2021) 13:2322. 10.3390/NU13072322 34371832 PMC8308813

[4] di Corcia M Tartaglia N Polito R Ambrosi A Messina G Francavilla VC. Functional properties of meat in athletes&rsquo; performance and recovery. Int J Environ Res Public Health. (2022) 19:5145. 10.3390/IJERPH19095145 35564540 PMC9102337

[5] Meier T Christen O. Gender as a factor in an environmental assessment of the consumption of animal and plant-based foods in Germany. Int J Life Cycle Assess. (2012) 17:550–64. 10.1007/S11367-012-0387-X

[6] Jeukendrup A Cronin L. Nutrition and elite young athletes. Med Sport Sci. (2011) 56:47–58. 10.1159/000320630 21178366

[7] Reinaldo JM Silva da DG Matos RC Leite MMR Mendes-Netto RS. Inadequação nutricional na dieta de atletas adolescentes. ABCS Health Sci. (2016) 41:156–62. 10.7322/ABCSHS.V41I3.905

[8] Pendrill F Persson UM Godar J Kastner T Moran D Schmidt S. Agricultural and forestry trade drives large share of tropical deforestation emissions. Global Environ Change. (2019) 56:1–10. 10.1016/J.GLOENVCHA.2019.03.002

[9] Barbosa C. Pecuária é responsável por 80% do desmatamento. Belém do Pará: Brasil de Fato. (2019). Available online at: https://www.brasildefato.com.br/2019/09/05/pecuaria-e-responsavel-por-80-do-desmatamento-na-amazonia-afirma-pesquisadora (accessed August 4, 2022).

[10] Garzilho J. Alimentação E Seus Impactos Ambientais: Abordagens Dos Guias Alimentares Nacionais E Estudos Da Dieta Dos Brasileiros. [Thesis (doctorate)]. São Paulo: Faculdade de Saúde Pública da Universidade de São Paulo (2019).

[11] IPCC- Intergovernmental Panel on Climate Change. Climate Change 2007: Mitigation. Contribution of Working Group III to the Fourth Assessment Report of the Intergovernmental Panel on Climate Change Cambridge University Press, Cambridge, United Kingdom and New York, NY, USA. (2007). Available online at: https://www.ipcc.ch/report/ar4/wg3/ (accessed August 4, 2022).

[12] IPCC- Intergovernmental Panel on Climate Change. Special Report on Climate Change, Desertification, Land Degradation, Sustainable Land Management, Food Security, and Greenhouse Gas Fluxes in Terrestrial Ecosystems. Intergovernmental Panel on Climate Change Geneva, Switzerland. (2019). Available online at: https://www.ipcc.ch/srccl/ (accessed August 8, 2022).

[13] WWP - World Wildlife Fund. One Planet Plate 2021 - Criteria and Background. World Wildlife Fund. (2021). Available online at: https://wwwwwfse.cdn.triggerfish.cloud/uploads/2022/05/one-planet-plate-criteria-2021_final.pdf (accessed August 8, 2022).

[14] Romaguera M Hoekstra AY Su Z Krol MS Salama MS. Potential of using remote sensing techniques for global assessment of water footprint of crops. Remote Sensing. (2010) 2:1177–96. 10.3390/RS2041177

[15] Feng K Siu YL Guan D Hubacek K. Assessing regional virtual water flows and water footprints in the yellow river Basin, China: a consumption based approach. Appl Geograp. (2012) 32:691–701. 10.1016/J.APGEOG.2011.08.004

[16] Sobhani SR Rezazadeh A Omidvar N Eini-Zinab H. Healthy diet: a step toward a sustainable diet by reducing water footprint. J Sci Food Agric. (2019) 99:3769–75. 10.1002/JSFA.9591 30637755

[17] Willett W Rockström J Loken B Springmann M Lang T Vermeulen S. Food in the anthropocene: the EAT–lancet commission on healthy diets from sustainable food systems. Lancet. (2019) 393:447–92. 10.1016/S0140-6736(18)31788-4 30660336

[18] Tucci M Martini D del Bo' C Marino M Battezzati A Bertoli S Porrini M Riso P. An Italian-mediterranean dietary pattern developed based on the EAT-lancet reference diet (EAT-IT): a nutritional evaluation. Foods. (2021) 10:558. 10.3390/FOODS10030558 33800396 PMC8002105

[19] Lynch H Johnston C Wharton C. Plant-based diets: considerations for environmental impact, protein quality, and exercise performance. Nutrients. (2018) 10:1841. 10.3390/NU10121841 30513704 PMC6316289

[20] Reguant-Closa A Harris MM Lohman TG Meyer NL. Validation of the athlete's plate nutrition educational tool: phase I. Int J Sport Nutr Exerc Metab. (2019) 29:628–35. 10.1123/IJSNEM.2018-0346 31141408

[21] Reguant-Closa A Roesch A Lansche J Nemecek T Lohman TG Meyer NL. The environmental impact of the athlete's plate nutrition education tool. Nutrients. (2020) 12:1–27. 10.3390/NU12082484 32824745 PMC7468909

[22] Passariello CL Marchionni S Carcuro M Casali G Pasqua A. della Hrelia S Malaguti M Lorenzini A. The mediterranean athlete's nutrition: are protein supplements necessary? Nutrients. (2020) 12:1–10. 10.3390/NU12123681 33260293 PMC7759839

[23] Scarano FR Silva JMC da. Production and international trade: challenges for achieving targets 6 and 11 of the global strategy for plant conservation in Brazil. Rodriguésia. (2018) 69:1577–85. 10.1590/2175-7860201869408

[24] IBGE- Instituto Brasileiro de Geografia e Estatística. Pesquisa de Orçamentos Familiares 2017 - 2018. IBGE, Coordenação de Trabalho e Rendimento. (2019). Available online at: https://biblioteca.ibge.gov.br/visualizacao/livros/liv101670.pdf (accessed August 6, 2022).

[25] de Onis M Onyango AW Borghi E Siyam A Nishida C Siekmann J. Development of a WHO growth reference for school-aged children and adolescents. Bull World Health Organ. (2007) 85:660–7. 10.2471/BLT.07.043497 18026621 PMC2636412

[26] Barufaldi LA de Azevedo Abreu G Oliveira JS dos Santos DF Fujimori E Vasconcelos SML. prevalência de comportamentos alimentares saudáveis em adolescentes brasileiros. Rev Saude Publica. (2016) 50:1–9s. 10.1590/S01518-8787.2016050006678 26982957 PMC4772693

[27] Pinheiro ABV Lacerda EM de A Benzecry EH Gomes MC da S Costa VM da. Tabela Para Avaliação De Consumo Alimentar Em Medidas Caseiras. 4th ed. São Paulo: Atheneu (2004).

[28] Garzillo JMF Machado PP Louzada ML da C Levy RB Monteiro CA. Pegadas dos alimentos e das preparações culinárias consumidos no Brasil. Pegadas Dos Alimentos E Das Preparações Culinárias Consumidos No Brasil (2019). São Paulo: Faculdade de Saúde Pública da USP.

[29] Freedman DS Ogden CL Blanck HM Borrud LG Dietz WH. The abilities of body mass index and skinfold thicknesses to identify children with low or elevated levels of dual-energy X-ray absorptiometry-determined body fatness. J Pediatr. (2013) 163:160-6.e1. 10.1016/J.JPEDS.2012.12.093 23410599 PMC4594849

[30] Marcelo de Queiroz Miranda J Vinícius Palmeira M Felipe Tubagi Polito L Regina Ferreira Brandão M Sales Bocalini D José Figueira Junior A. Prevalência de sobrePeso e obesidade infantil em instituições de ensino: públicas vs. privadas. Rev Bras Med Esporte. (2015) 2:34–50. 10.1590/1517-869220152102143660

[31] Sotoriva EMS Miraglia F. Análise De Hábitos Alimentares E Conhecimento Nutricional De Adolescentes Atletas De Judô. Hígia-Revista De Ciências Da Saúde E Sociais Aplicados Do Oeste Baiano. (2017). Available online at: http://fasb.edu.br/revista/index.php/higia/article/view/180 (accessed August 4, 2022).

[32] Poortmans JR Carpentier A Pereira-Lancha LO Lancha A. Protein turnover, amino acid requirements and recommendations for athletes and active populations. Braz J Med Biol Res. (2012) 45:875–90. 10.1590/S0100-879X2012007500096 22666780 PMC3854183

[33] Moore DR. Nutrition to Support Recovery from Endurance Exercise: Optimal Carbohydrate and Protein Replacement. Curr Sports Med Rep. (2015) 14:294–300. 10.1249/JSR.0000000000000180 26166054

[34] Lassen AD Christensen LM Trolle E. Development of a danish adapted healthy plant-based diet based on the EAT-lancet reference diet. Nutrients. (2020) 12:738. 10.3390/NU12030738 32168838 PMC7146415

[35] Perignon M Vieux F Soler LG Masset G Darmon N. Improving diet sustainability through evolution of food choices: review of epidemiological studies on the environmental impact of diets. Nutr Rev. (2017) 75:2. 10.1093/NUTRIT/NUW043 27974596 PMC5155614

[36] Conceição Pereira da. Fonseca M da Salay E. Beef, chicken and pork consumption and consumer safety and nutritional concerns in the City of Campinas, Brazil. Food Control. (2008) 19:1051–8. 10.1016/J.FOODCONT.2007.11.003

[37] Souza A de M Pereira RA Yokoo EM Levy RB Sichieri R. Alimentos mais consumidos no Brasil: Inquérito Nacional de Alimentação 2008-2009. Rev Saude Publica. (2013) 47:190s−9s. 10.1590/S0034-89102013000200005 23703263

[38] Brasil. Guia Alimentar Para A População Brasileira. 2nd ed. Brasília: Ministério da Saúde (2014). 156p.

[39] Whitnall T Pitts N. Global trends in meat consumption. Agric Commod. (2019) 9:96–9. Available online at: https://search.informit.org/doi/10.3316/informit.309517990386547

[40] Bursztyn M Eiró F. Mudanças Climáticas E Distribuição Social Da Percepção De Risco No Brasil. Revista Sociedade e Estado. (2015) 30:471–93.

[41] Ritchie H Reay DS Higgins P. The impact of global dietary guidelines on climate change. Global Environ Change. (2018) 49:46–55. 10.1016/j.gloenvcha.2018.02.005

[42] Garzillo JMF Machado PP Leite FHM Steele EM Poli VFS Louzada ML da C. Carbon footprint of the Brazilian diet. Rev Saude Publica. (2021) 55:90. 10.11606/S1518-8787.2021055003614 34910024 PMC8621484

[43] Heller MC Willits-Smith A Meyer R Keoleian GA Rose D. Greenhouse gas emissions and energy use associated with production of individual self-selected US diets. Environ Res Lett. (2018) 13:044004. 10.1088/1748-9326/AAB0AC 29853988 PMC5964346

[44] Travassos GF Antônio da. Cunha D Coelho AB. The environmental impact of Brazilian adults' diet. J Clean Prod. (2020) 272:122622. 10.1016/J.JCLEPRO.2020.12262233353574

[45] Arrieta EM González AD. Impact of current, national dietary guidelines and alternative diets on greenhouse gas emissions in Argentina. Food Policy. (2018) 79:58–66. 10.1016/J.FOODPOL.2018.05.003

[46] Temme EH Toxopeus IB Kramer GF Brosens MC Drijvers JM Tyszler M. Greenhouse gas emission of diets in the Netherlands and associations with food, energy and macronutrient intakes. Public Health Nutr. (2015) 18:2433–45. 10.1017/S1368980014002821 25543460 PMC10271514

[47] Colombo PE Elinder LS Lindroos AK Parlesak A. Designing nutritionally adequate and climate-friendly diets for omnivorous, pescatarian, vegetarian and vegan adolescents in Sweden using linear optimization. Nutrients. (2021) 13:2705. 10.3390/NU13082507/S1 34444667 PMC8398609

[48] Aiking H de Boer J. The next protein transition. Trends Food Sci Technol. (2020) 105:515. 10.1016/J.TIFS.2018.07.008 PMC7127173 38620223

[49] Nebl J Haufe S Eigendorf J Wasserfurth P Tegtbur U Hahn A. Exercise capacity of vegan, lacto-ovo-vegetarian and omnivorous recreational runners. J Int Soc Sports Nutr. (2019) 16:23. 10.1186/s12970-019-0289-4 31109329 PMC6528342

[50] Katharina CW. Vegan diet in sports and exercise – health benefits and advantages to athletes and physically active people: a narrative review. Int J Sports Exerc Med. (2020) 6:165. 10.23937/2469-5718/1510165

[51] Meyer NL Reguant-Closa A Nemecek T. Sustainable diets for athletes. Curr Nutr Rep. (2020) 9:147–62. 10.1007/S13668-020-00318-0 32504413

